# Systemic Inflammatory Score predicts Overall Survival in patients with Cervical Cancer

**DOI:** 10.7150/jca.56170

**Published:** 2021-04-30

**Authors:** Mu Xu, Qibin Wu, Liangzhi Cai, Xiaoqi Sun, Xiaoyan Xie, Pengming Sun

**Affiliations:** 1 Department of Gynecology, Fujian Maternity and Child Health Hospital, Affiliated Hospital of Fujian Medical University, Fuzhou, China.; 2 Laboratory of Gynecologic Oncology, Fujian Maternal and Child Health Hospital, Affiliated Hospital of Fujian Medical University, Fuzhou, China.

**Keywords:** cervical cancer, systemic inflammation score, lymphocyte-to- monocyte ratio, albumin, prognosis

## Abstract

**Background:** To evaluate the prognostic value of the systemic inflammatory score (SIS) in cervical cancer patients.

**Methods:** A total of 264 patients with FIGO stage (2009) IB-IIA cervical cancer undergoing radical resection from January 2014 to December 2017 were recruited. The optimal cutoff values for inflammatory biomarkers were calculated by X-tile software. The prognostic factors were investigated using univariate and multivariate Cox analyses. Time-dependent receiver operating characteristic (time-ROC) analysis and the concordance index (C-index) were used to compare the prognostic impact of factors.

**Results:** In total, 264 patients with cervical cancer were included in the study. The optimal cutoff value for lymphocyte-to-monocyte ratio (LMR) was 4.1. In multivariate analysis, FIGO stage, lymphovascular invasion, lymph node metastasis, preoperative serum albumin (Alb), and LMR were independent prognostic factors (P<0.05). Then, we combined preoperative Alb and LMR to establish the SIS. Multivariate analysis showed that the SIS was an independent factor that affected survival (P<0.05). When stratified by FIGO stage, significant differences in survival were also found for patients with different SISs (P<0.05). When the SIS and FIGO stage were combined, the time-ROC curve was superior to that of FIGO stage only. The C-index of the model combining the SIS and FIGO stage was 0.786 (95% CI 0.699-0.873), which was significantly higher than that of the model with FIGO stage only (0.676, 95% CI 0.570-0.782, P=0.0049).

**Conclusions:** The preoperative SIS is a simple and useful prognostic factor for postoperative survival in patients with cervical cancer. It might assist in the identification of high-risk patients among patients with the same FIGO stage.

## Introduction

Cervical cancer (CC) remains the most common gynaecologic malignancy in females worldwide 1-2. The postoperative recurrence and metastasis are the main causes of death in CC 3-4. The International Federation of Gynaecology and Obstetrics (FIGO) staging system is the main criterion used to predict the prognosis of patients with CC so far. However, the prognosis varies in CC patients with the same FIGO stage. Some patients with early stage disease experience recurrence in a short time after surgery, leading to poor prognosis 5. Therefore, it is necessary to find more potential biomarkers in clinical practice to improve prognostic prediction.

Previous studies showed inflammation affected cancer pathogenesis and progression 6-7. Systemic inflammatory biomarkers, such as the lymphocyte-to-monocyte ratio (LMR), neutrophil-to-lymphocyte ratio (NLR), and platelet-to-lymphocyte ratio (PLR), have been reported as prognostic factors in various tumours 8-10. The levels of serum albumin (Alb) before operation have also been reported as an independent predictor of prognosis in patients with malignancies 11. These biomarkers can be detected in an easy and convenient way. Recently, the systemic inflammatory score (SIS), based on the level of preoperative Alb and LMR, has been found to be prognostic factor in some cancers 12-13. However, the effect of SIS on the prognosis of CC patients remains unknown. Therefore, we aimed to assess the prognostic value of the SIS for patients with CC who underwent radical surgery.

## Materials and Methods

### Patients

A total of 264 patients with cervical cancer undergoing radical resection at the Fujian Provincial Maternity and Children's Hospital from January 2014 to December 2017 were recruited. The following inclusion criteria were applied: (1) histologically confirmed cervical cancer; (2) FIGO stage (2009) IB-IIA with no evidence of tumours invading adjacent organs or distant metastasis; and (3) radical hysterectomy with pelvic lymphadenectomy. The study excluded patients who had distant metastasis in the liver, lung, or peritoneum/pelvic cavity diagnosed before or during the operation; who underwent preoperative neoadjuvant radiotherapy and/or chemotherapy; who had active infection or inflammatory diseases within a month before blood examination; or who had incomplete/inaccurate medical records. The FIGO 2009 clinical staging system was used for tumour staging. This retrospective study was approved by the Ethics Committee of the Fujian Provincial Maternity and Children's Hospital. Written informed consent was obtained from all participants.

### Definition of systemic inflammatory biomarkers

The blood testing was carried out within 1 week before surgery, including the neutrophil count, lymphocyte count, platelet count, and Alb level. LMR was defined as the total number of lymphocytes divided by the total number of monocytes. NLR and PLR were defined as the total number of neutrophils or platelets divided by the total number of lymphocytes. The cutoff value of Alb was 40 g/l 12-13. The optimal cutoff values for LMR, NLR and PLR were calculated by the X-tile software (Yale University, New Haven, CT, USA) 14.

### Follow‑up

Patients underwent follow-up examinations every 3 months for years 1 and 2, then every 6 months during years 3-5. The final follow-up evaluation was conducted in December 2019. The median follow-up period was 44 months (range 3-72 months). The main examinations included physical examination, vaginal examination, laboratory testing (including cancer antigen 125 (Ca125), Squamous cell carcinoma antigen (SCC)), chest radiography, and pelvic ultrasonography. The lung computed tomography (CT) or pelvic magnetic resonance imaging (MRI) scan was performed when necessary. Overall survival (OS) was defined as the time from surgery to the date of death or to the last follow-up.

### Statistical analysis

Statistical analyses were performed using SPSS for Windows version 18.0 (SPSS Inc., Chicago, IL, USA) and R ver. 3.6.2 (R Foundation for Statistical Computing, Vienna, Austria). Categorical and continuous variables were compared using a chi-square test or Fisher's exact test and a *t* test, respectively. The survival curves were calculated by the Kaplan‑Meier method, and differences between them were examined using the log-rank test. Variables associated with *P* <0.05 in univariate analysis were then included in the multivariate Cox regression analysis. The time-dependent receiver operating characteristic (time-ROC) curves, which was an extension of the ROC curve 15, were used to compare the prognostic abilities of the scores. Model performance was then assessed by Concordance indices (C-index). The time-ROC analysis and C-index were performed respectively by using the R packages “rms” and “time ROC”. All tests were 2-sided, and P < 0.05 indicated that the difference was statistically significant.

## Results

### Patient characteristics

The optimal cutoff levels for LMR, NLR and PLR were 4.1, 1.8 and 132.8, respectively, which were calculated by X-tile software (Supplemental Fig. 1).

In total, 264 patients were included in the study. The clinicopathological features of the patients were shown in Table 1. The median age of the patients was 47 years (24-71 years). Based on the FIGO staging system, 177 (67.0%), 37 (14.1%), and 50 (18.9%) of the patients had FIGO stage Ib1, Ib2, and IIa disease, respectively. According to the cutoff value, 120 (45.5%) patients had a high LMR, 144 (54.5%) had a low LMR, 133 (50.4%) had a high NLR, 131 (49.6%) had a low NLR, 128 (48.5%) had a high PLR, 136 (51.5%) had a low PLR, 155 (58.7%) had a high Alb and 109 (41.3%) had a low Alb (Table 1).

### Survival analysis

The 5-year OS rate was 89.1% in all patients. Univariate analysis showed that FIGO stage, lymphovascular invasion, deep stromal invasion, lymph node metastasis, preoperative Alb, and inflammatory indicators including LMR, NLR, and PLR were associated with OS (all *P*<0.05). Further multivariate Cox regression analysis showed that FIGO stage, lymphovascular invasion, lymph node metastasis, preoperative Alb and LMR were independent factors for prognosis (Table 1).

### Correlations between clinicopathological features and the SIS

As Alb level (≥40 g/l) and LMR ≥4.1 were associated with good OS (both *P* < 0.05, Table 1), the new indicator SIS was then established based on preoperative Alb and LMR, and the patients were divided into three subgroups. Patients with both decreased serum albumin and decreased LMR (<40 g/l and <4.1, respectively) were assigned a score of 2; patients with either decreased serum albumin or decreased LMR were assigned a score of 1; and patients with both elevated serum albumin and elevated LMR (≥40 g/l and ≥4.1, respectively) were assigned a score of 0.

The relationships between clinicopathological factors and the SIS are shown in Table 2. The results showed that higher FIGO stage, lymphovascular invasion, deep stromal invasion, and lymph node metastasis were significantly associated with higher SISs (all *P* < 0.05, Table 2).

### Influence of the SIS on OS

**Figure 1:**
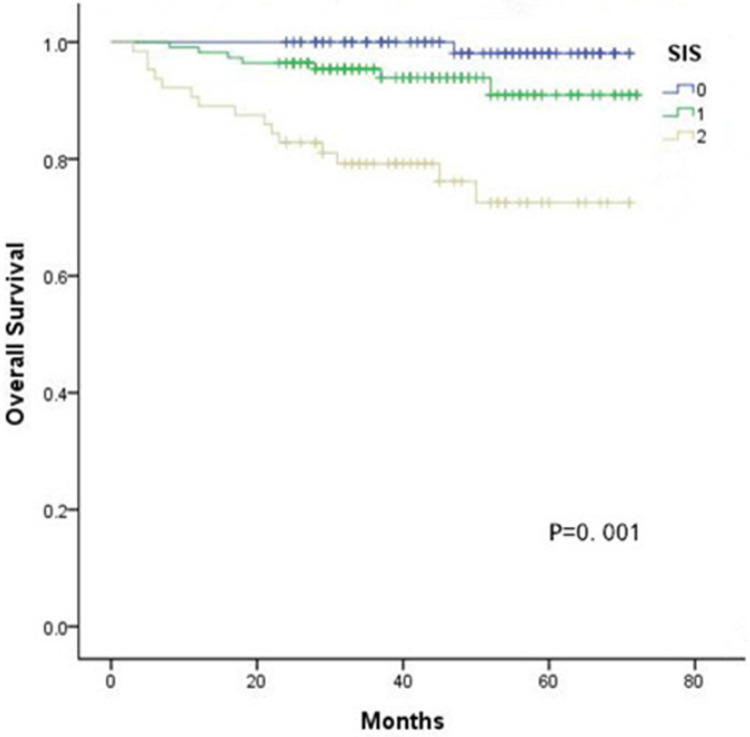
Kaplan-Meier analysis of OS of CC patients according to the SIS.

**Figure 2:**
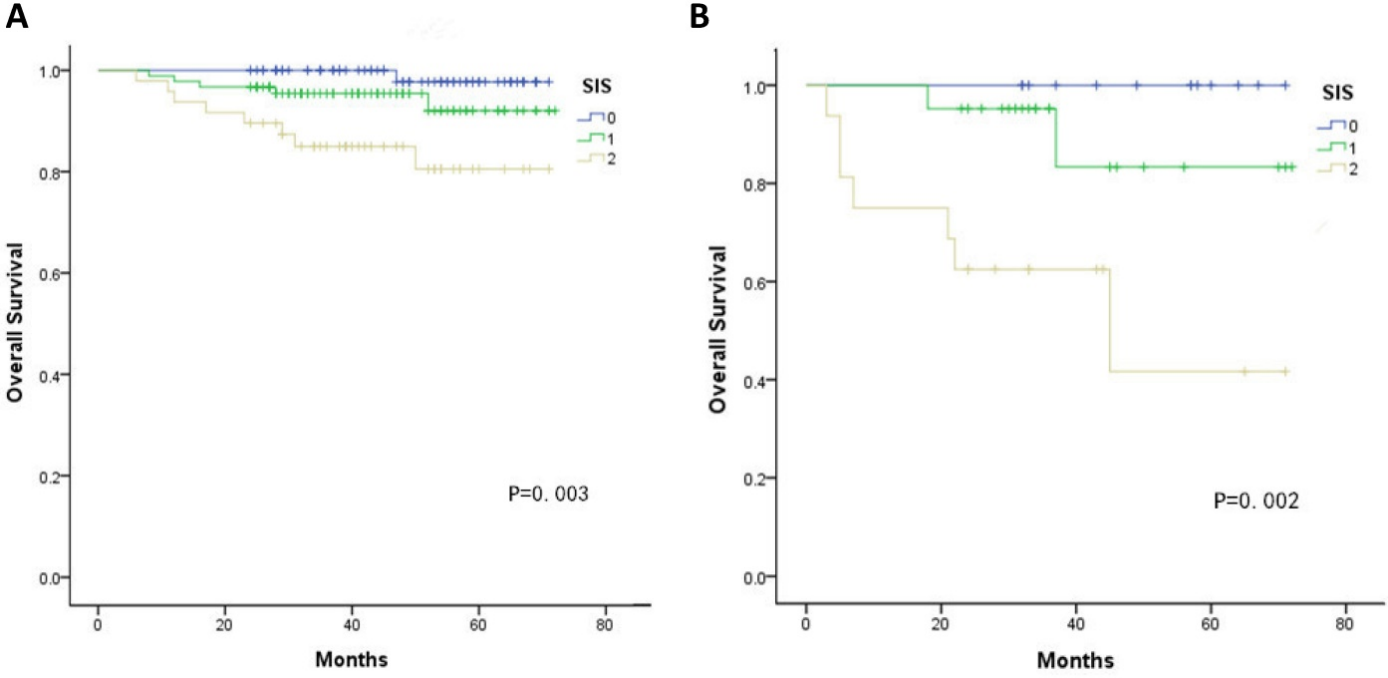
** A.** Association of the SIS with the OS of patients with FIGO stage Ib **B.** Association of the SIS with the OS of patients with FIGO stage IIa.

Kaplan-Meier curves showed significant differences in the 5-year OS among three groups according to the SIS (SIS=0: 98.1%, SIS=1: 72.5%, SIS=2: 72.5%, all *P* < 0.05, Fig. 1). Furthermore, multivariate analyses revealed that FIGO stage, lymphovascular invasion, deep stromal invasion and SIS were significantly associated with OS (all *P* < 0.05, Table 3). In addition, when stratified by FIGO stage, there were also significant differences in OS with a SIS of 0, 1 and 2 in the stage Ib and II subgroups (all *P* < 0.05, Fig. 2).

### The predictive value of SIS on prognosis

**Figure 3:**
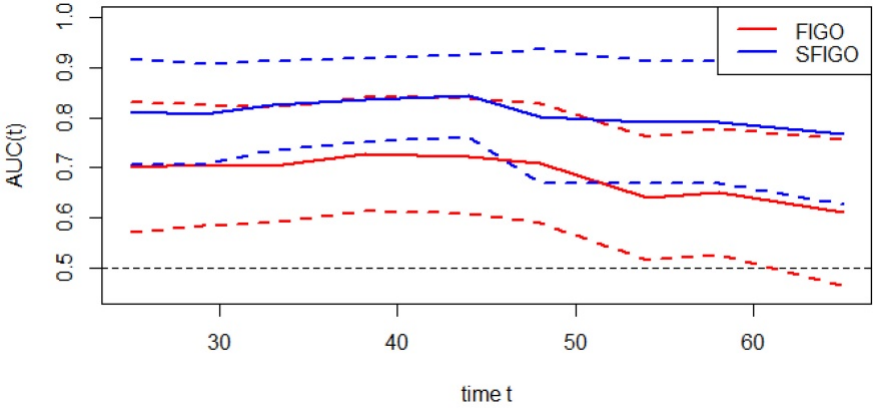
Time-dependent ROC curves of FIGO stage only and new model (FIGO stage and SIS, SFIGO) for the prediction of OS. Red and blue solid lines represent the estimated AUCs for FIGO stage only and new model (SFIGO), respectively. Broken lines represent the 95% confidence intervals for each AUC. OS indicates Overall Survival.

Moreover, the predictive accuracy of the model combining the SIS and FIGO stage (SFGO) was compared with that of the model containing FIGO stage only by establishing time-ROC curves (Fig. 3). As the results showed, the time-ROC curve of the model combining the SIS and FIGO stage was superior to that of the model containing FIGO stage only. In addition, the C-index of the model combining the SIS and FIGO stage was 0.786 (95% CI: 0.699-0.873), which was significantly higher than that of the model with only FIGO stage (0.676, 95% CI: 0.570-0.782; *P*=0.0049).

## Discussion

Although FIGO stage is the most important clinical prognostic indicator for cervical cancer patients, many patients with the same FIGO stage have different treatment outcomes due to tumour heterogeneity 5. Therefore, it is necessary to find other indicators to assist in predicting the prognosis of cervical cancer. As Virchow originally made links between cancer and inflammation in 1863 16, more and more investigations have revealed that inflammation plays an important role in tumour progression and metastasis 7, 17. Previous studies have also shown that inflammatory indicators significantly influence the prognosis of gynaecologic cancers 18-20. Recently, the SIS, based on the combination of serum albumin and LMR, was reported to have prognostic value in renal cell carcinoma and colorectal cancer 12, 13. Compared to other single nutritional or inflammatory makers, the SIS may be a better indicator because of considering the influences of both nutritional condition and inflammation on the prognosis of tumours. However, the influence of the SIS on the prognosis of cervical cancer remains unclear.

The optimal cutoff point of the inflammatory factors obtained varied in previous studies such as ROC curve, quartiles 20. In contrast, the X-tile software is a new method for obtaining the optimal cutoff through time-dependent cutoff value analysis based on survival information. Then the cutoff value was identified with minimum P values from log-rank χ2 statistics for the categorical biomarkers in terms of survival 14. Compared with the ROC curve method merely based on the outcome, the use of the X-tile software seems more appropriate. The method had been widely used in other studies 21, 22. In this study, the cutoff value of LMR was 4.1, as calculated by X-tile. Multivariable analysis demonstrated that LMR ≥4.1 and Alb ≥40 g/l were associated with favourable OS. Additionally, the SIS was associated with a number of variables that were previously shown to be predictive of poor outcomes. The SIS was also an independent prognostic factor according to multivariable analysis.

LMR consists of lymphocytes and monocytes. As the basic components of the immune system, lymphocytes can stimulate the proliferation of cytotoxic T cells by secreting cytokines such as IFN-γ and TNF-α. As a result, the tumour cell proliferation, invasion and metastasis are inhibited, resulting in cytotoxic death and improving the prognosis 23, 24. Moreover, the lymphocytes transform into tumour-infiltrating lymphocytes when migrate to the tumour microenvironment, resulting in anti-tumour activity and the inhibition of angiogenesis to improve the outcomes in cancers 25. Thus, a decline in lymphocytes predicted poor outcomes in patients with tumour 26. In addition, monocytes can be recruited to tumour tissues and differentiate into tumour-associated macrophages (TAMs). Then, TAMs release epidermal growth factor and angiogenic factors to promote tumour cell proliferation and migration 27-29. Chen et al 30 demonstrated the prognostic value of preoperative LMR in predicting the survival of 485 patients with FIGO stage IB1-IIA cervical cancer. Therefore, LMR plays an important role in the prognosis of cervical cancer. Alb is closely related to nutritional status in patients, which is a good indicator of immune status. Zheng found that hypoalbuminemia was associated with poor survival in 798 patients with early-stage cervical cancer 31, which was also observed in our study. Additionally, the SIS was also significantly influenced by prognosis when stratified by FIGO stage. In agreement with previous findings, we demonstrated that high SIS was an independent predictor of diminished survival for CC patients.

Recently, time-ROC analysis has been used in some studies 14. It's a method that applies ROC curve analysis to time-dependent variables. The advantage of this method is that it enables the analysis of survival data with censoring using ROC curves, which is a popular method of determining sensitivity and specificity. What's more, the significant difference among prognostic factors can be assessed visually. In our study, we created a new prognostic model by combining the SIS and FIGO stage. As the result showed, the time-ROC curve and the C-index of the new model combining the SIS with FIGO stage were significantly higher than the model with traditional FIGO stage only, which suggested that the predictive accuracy of the new model was better. In other words, the SIS could improve the accuracy of the prognostic assessment in patients with cervical cancer. Therefore, the SIS can be used as a supplement to FIGO stage in preoperative risk stratification to improve the prediction of clinical outcomes.

However, there were also some limitations in the study. First, as a retrospective, single-center study, the possibility of selection bias is inevitable. Therefore, a large-scale prospective validation study is required to validate the results. Second, the results of this study are not suitable for cervical cancer patients after neoadjuvant therapy.

## Conclusions

Nevertheless, this study found that the preoperative SIS is a novel and simple prognostic factor for cervical cancer. In clinical practice, the SIS could be considered as a supplement to the FIGO stage system and assist in the identification of high-risk patients among patients with the same FIGO stage.

## Supplementary Material

Supplementary figure S1.

## Figures and Tables

**Table 1:** Correlations between clinicopathologic variables and OS in univariate and multivariate analysis

Clinicopathological features	No. of patients	Univariate analysis		Multivariate analysis	
		HR (95%CI)	*P*	HR (95%CI)	*P*
**Age (y)**			0.513		-
<45	106	Reference			
≥45	158	1.332 (0.564-3.145)			
**FIGO stage**			0.008		0.022
IB1	177	Reference		Reference	
IB2	37	2.935 (0.982-8.77)		0.232 (0.076-0.709)	
IIa	50	4.146 (1.642-10.467)		0.313 (0.099-0.984)	
**Surgery**			0.485		-
Open	134	Reference			
Lap	130	0.484 (0.565-3.320)			
**Histological type**			0.658		-
SCC	203	Reference			
AC	46	0.751 (0.221-2.55)			
Others	15	1.726 (0.399-7.461)			
**Lymphovascular invation**			0.001		0.041
No	182	Reference		Reference	
Yes	82	5.697 (2.34-13.875)		3.01 (1.045-8.671)	
**Depth of invasion**			0.011		0.815
<1/2	140	Reference		Reference	
≥1/2	124	3.337 (1.315-8.463)			
**Lymph node metastasis**			0.002		0.024
No	228	Reference		Reference	
Yes	36	3.901 (1.653-9.209)		0.359 (0.158-0.703)	
**Alb (g/l)**			0.026		0.039
<40	109	Reference		Reference	
≥40	155	0.194 (0.07-0.499)		0.123-0.945	
**LMR**			0.014		0.020
<4.1	144	Reference		Reference	
≥4.1	120	0.111 (0.026-0.471)		0.167 (0.037-0.755)	
**NLR**			0.019		0.400
<1.8	131	Reference		Reference	
≥1.8	133	3.055 (1.204-7.752)		1.542 (0.562-4.231)	
**PLR**			0.024		0.135
<132.8	136	Reference		Reference	
≥132.8	128	2.783 (1.143-6.778)		2.134 (0.789-5.771)	

Abbreviations: OS, overall survival; Alb, albumin; LMR, lymphocyte-to- monocyte ratio; NLR, neutrophil-to-lymphocyte ratio; PLR, platelet-to-lymphocyte ratio; SCC, squamous cell carcinoma; AC, adenocarcinoma.

**Table 2:** Relationship between the SIS and clinicopathological variables of patients

Clinicopathological features	SIS			*P* value
	0	1	2	
**Age (y)**				0.474
<45	35 (39.8%)	49 (43.8%)	22 (34.4%)	
≥45	53 (60.2%)	63 (56.2%)	42 (65.6%)	
**FIGO stage**				0.020
IB1	67 (76.1%)	76 (67.8%)	34 (53.1%)	
IB2	8 (9.1%)	15 (13.4%)	14 (21.9%)	
IIa	13 (14.8%)	21 (18.8%)	16 (25.0%)	
**Surgery**				0.191
Open	55 (62.5%)	50 (44.6%)	29 (45.3%)	
Lap	33 (37.5%)	62 (55.4%)	35 (54.7%)	
**Histological type**				0.493
SCC	70 (79.5%)	82 (73.2%)	51 (79.7%)	
AC	15 (17.1%)	23 (20.5%)	8 (12.5%)	
Others	3 (3.4%)	7 (6.3%)	5 (7.8%)	
**Lymphovascular invation**				<0.001
No	75 (85.2%)	73 (65.2%)	34 (53.1%)	
Yes	13 (14.8%)	39 (34.8%)	30 (46.9%)	
**Depth of invasion**				<0.001
<1/2	62 (70.5%)	54 (48.2%)	24 (37.5%)	
≥1/2	26 (29.5%)	58 (51.8%)	40 (62.5%)	
**Lymph node metastasis**				<0.001
No	86 (97.7%)	94 (83.9%)	48 (75.0%)	
Yes	2 (2.3%)	18 (16.1%)	16 (25.0%)	
**NLR**				0.002
<1.8	56 (63.6%)	52 (46.4%)	23 (35.9%)	
≥1.8	32 (36.4%)	60 (53.6%)	41 (64.1%)	
**PLR**				0.009
<132.8	56 (63.6%)	55 (49.1%)	25 (39.1%)	
≥132.8	32 (36.4%)	57 (50.9%)	39 (0.9%)	

Abbreviations: SIS, Systemic Inflammatory Score; SCC, squamous cell carcinoma; AC, adenocarcinoma; NLR, neutrophil-to-lymphocyte ratio; PLR, platelet-to-lymphocyte ratio.

**Table 3:** Multivariate analysis of clinicopathologic variables in relation to OS in patients

Clinicopathological features	No. of patients	OS	
		HR (95%CI)	*P* value
**FIGO stage**			0.027
IB1	177	Reference	
IB2	37	1.285 (0.375-4.403)	
IIa	50	4.053 (1.335-12.310)	
**Lymphovascular invation**			0.033
No	182	Reference	
Yes	82	3.092 (1.093-8.741)	
**Depth of invasion**			0.906
<1/2	140	Reference	
≥1/2	124	0.933 (0.295-2.956)	
**Lymph node metastasis**			0.025
No	228	Reference	
Yes	36	2.116 (1.014-5.746)	
**NLR**			0.352
<1.8	131	Reference	
≥1.8	133	1.632 (0.582-4.578)	
**PLR**			0.162
<132.8	136	Reference	
≥132.8	128	2.062 (0.747-5.90)	
**SIS**			0.008
0	88	Reference	
1	112	3.639 (0.421-31.433)	
2	64	11.733 (1.410-97.616)	

Abbreviations: OS, overall survival; SIS, Systemic Inflammatory Score; NLR, neutrophil-to- lymphocyte ratio; PLR, platelet-to-lymphocyte ratio.

## Abbreviations

OS: overall survival
SIS: Systemic Inflammatory Score
Alb: albumin
LMR: lymphocyte-to-monocyte ratio
NLR: neutrophil-to-lymphocyte ratio
PLR: platelet-to-lymphocyte ratio
SCC: squamous cell carcinoma
AC: adenocarcinoma
