# Supplementary material for: Systemic Inflammatory Score predicts Overall Survival in patients with Cervical Cancer
Source: J Cancer. 2021 Apr 30;12(12):3671–7. doi: 10.7150/jca.56170 (PMC8120179; doi:10.7150/jca.56170)
Supplement: Supplementary file 1 — Supplementary figure S1. [file jcav12p3671s1.pdf]

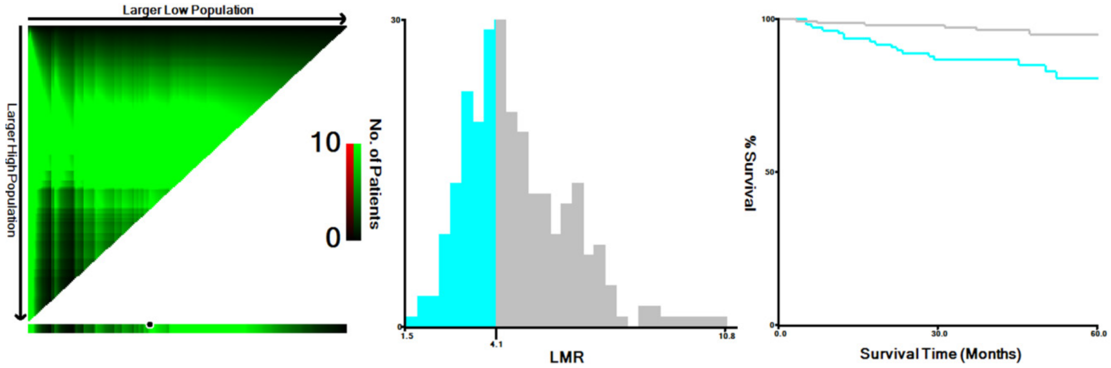

Supplemental Fig. 1 The cut-off value of LMR according to X-tile. LMR indicates lymphocyte-to-monocyte ratio.
